# Affective Touch in Preterm Infant Development: Neurobiological Mechanisms and Implications for Child–Caregiver Attachment and Neonatal Care

**DOI:** 10.3390/children11111407

**Published:** 2024-11-20

**Authors:** Valentina Lucia La Rosa, Alessandra Geraci, Alice Iacono, Elena Commodari

**Affiliations:** Department of Educational Sciences, University of Catania, 95124 Catania, Italy; alessandra.geraci@unict.it (A.G.); alice.iacono18@gmail.com (A.I.); e.commodari@unict.it (E.C.)

**Keywords:** affective touch, C-tactile fibers, neurodevelopment, attachment, preterm infants, skin-to-skin contact, kangaroo care

## Abstract

Background/Objectives: Affective touch is crucial in infant development, particularly in regulating emotional, cognitive, and physiological processes. Preterm infants are often deprived of essential tactile stimulation owing to their early exposure to the external environment, which may affect long-term developmental outcomes. This review aimed to examine the neurobiological mechanisms of affective touch and highlight effective interventions, such as skin-to-skin contact (SSC) and kangaroo care (KC), to promote development in preterm infants. Methods: This review summarizes recent studies in the literature on affective touch, the role of C-tactile fibers, and the effects of tactile interventions in neonatal care. Studies were selected based on their relevance to the care and development of preterm infants, with a focus on physiological and neurodevelopmental outcomes. Key interventions, including SSC and massage therapy, are discussed in relation to their effectiveness in the neonatal intensive care unit (NICU). Results: The results suggest that affective touch, mainly through activation of tactile C-fibers, improves caregiver–infant bonding, reduces stress responses, and supports neurodevelopment in preterm infants. Interventions such as SSC and KC have also been shown to improve physiological regulation in these infants, including heart rate, breathing, and temperature control while promoting emotional regulation and cognitive development. Conclusions: Affective touch is a key component of early development, particularly in preterm infants admitted to the NICU. Integrating tactile interventions such as SSC and KC into neonatal care practices may significantly improve long-term developmental outcomes. Future research should explore the epigenetic mechanisms underlying affective touch and further refine tactile interventions to optimize neonatal care.

## 1. Introduction

Within the caregiver–infant dyad, especially during the first months of life, a specific mode of communication is established, not so much “mind to mind” as “body to body” [1]. Recent studies have provided evidence for this specific type of communication, mediated by the body and based on physical contact, which characterizes early dyadic interactions and is essential for strengthening the attachment bond [2] and promoting the infant’s psychophysiological balance [1].

This physical and embodied communication is a multisensory experience that includes various forms of physical closeness such as holding, caressing, and hugging. These actions create a complex nonverbal dialogue between caregivers and infants, both of which contribute to establishing rhythmic cycles of co-regulation, an interpersonal process in which emotions, behaviors, and physiological states are synchronized [1]. Importantly, these embodied interactions are not just about calming or soothing, but also play a fundamental role in the infant’s developmental trajectory, particularly in forming a bodily self and self–other distinctions.

Central to this dynamic is *affective touch*, which activates C-tactile afferents, specialized nerves tuned to gentle and caressing touch. This form of touch triggers activity in the insular cortex, a brain region associated with affective processing and social bonding, thereby supporting the emotional regulation and attachment processes. Research has shown that early touch experiences help shape infants’ ability to recognize their bodies, which is critical for developing bodily self-awareness, a precursor to more complex cognitive and social skills [3,4].

Moreover, the cycles of attunement and misattunement, that naturally occur in these body-to-body exchanges, play a crucial role. Misattunement moments, when caregivers and infants are out of sync, provide opportunities for “reparation,” where the dyad can restore synchrony. This process of “embodied reparation” not only strengthens the bond but also promotes resilience and self-regulatory capacities in infants. Repairing disruptions and returning to a state of attunement through physical touch and proximity are essential to building an infant’s emotional and physiological stability [1,5].

For preterm infants, who are often deprived of typical early physical exchanges with primary caregivers due to prolonged stays in the Neonatal Intensive Care Unit (NICU) and necessary medical interventions, these opportunities for affective contact are significantly reduced. This lack of early tactile experience may have long-term effects on the physiological, emotional, and social development of these children. Therefore, understanding how affective touch, including interventions such as *skin-to-skin contact,* can address these developmental challenges is critical for optimizing their developmental outcomes.

In light of these considerations, this review highlights the critical role of early physical interactions between caregivers and infants with a focus on affective touch in the context of preterm birth. We carefully selected all sources from indexed journals published by renowned publishers that met rigorous peer-review standards, ensuring the quality and scientific rigor of each source. By reviewing key scientific findings, we demonstrated how these early interactions shape the developmental trajectories of term and preterm infants. We also explored the practical implications of these findings, showing how they can guide care practices, early interventions, and health policies. Recognizing the importance of affective contact in the care of preterm infants offers valuable insights for optimizing therapeutic approaches, improving emotional and physiological well-being, and supporting long-term positive developmental outcomes in these vulnerable infants.

## 2. Early Tactile Interactions and Infant Development

Touch, one of the earliest human senses to develop, appears as early as the eighth week of pregnancy [6]. This fundamental sense comprises two primary dimensions: a *sensory-discriminative dimension*, which allows individuals to localize stimuli on the skin and judge their intensity, and a *motivational–affective dimension*, which shapes the emotional experience of touch [7]. The sensory-discriminative dimension is essential for object exploration, manipulation, and motor development. Through tactile exploration, children learn to discriminate texture, pressure, and spatial properties, which form the basis for more complex motor actions such as grasping and manipulating objects [8]. This process is crucial to cognitive development, enabling children to map their environment and interact with the physical world.

In addition to its role in motor and cognitive development, touch is fundamental to early caregiver–infant interactions. Early tactile exchanges between caregiver and infant, such as holding, stroking, and rocking, help regulate the infant’s physiological and emotional states [9,10]. These interactions lay the foundation for *co-regulation*, a process in which the caregiver helps the child manage stress and maintain emotional stability through physical contact [5]. Tactile interactions are particularly effective in supporting the child’s development of self-regulation, a skill that underlies the child’s ability to manage physiological and emotional responses to external stimuli [11]. In particular, some research has shown that consistent tactile stimulation in early childhood is associated with more stable heart rate variability, lower cortisol levels, and better overall stress management [12].

Therefore, touch is crucial in creating social bonds and communicating with others. The “skin as social organ” hypothesis suggests that skin is not only a protective barrier but also actively participates in nonverbal communication [13]. In fact, through tactile exchange, caregivers and infants engage in a form of nonverbal dialogue that is essential for emotional development and attachment formation. This physical communication is an integral part of human interaction and allows for the exchange of emotions such as comfort, empathy, and reassurance, which are fundamental to a child’s emotional well-being and sense of security [14].

Early tactile interactions are frequent and constant for term infants, allowing for appropriate emotional and physiological regulation. These infants benefit from immediate skin-to-skin contact after birth, which promotes the regulation of body temperature, heart rate, and stress hormones and helps establish a secure attachment to the caregiver [15]. The constant provision of tactile stimulation to term infants also promotes the maturation of the nervous system, facilitating the development of motor skills, sensory processing, and cognitive mapping of the environment [16].

In contrast, preterm infants face significant difficulties in receiving early tactile stimulation due to the medicalized environment of the NICU, where they may be separated from their caregivers and subjected to invasive medical procedures [17]. These infants often do not have the opportunity for regular skin-to-skin contact, which is critical for regulating physiological and emotional states [18]. Research shows that premature infants deprived of early tactile stimulation are at increased risk for developmental delays, emotional dysregulation, and stress-related disorders [19]. Furthermore, preterm birth significantly impacts the infant’s ability to perceive and process tactile stimuli. The somatosensory system, which begins to develop in utero, is often underdeveloped in preterm infants, resulting in impaired tactile sensitivity and response. Preterm infants are less able to process sensory information, affecting their ability to respond to physical contact and engage in affective interactions [14]. Some studies suggest that preterm infants may be more sensitive to tactile stimuli than full-term infants. Andrè et al. [20] found that almost all preterm infants could detect a very light mechanical stimulus (0.008 g), while term infants showed a lesser response. This increased sensitivity may be due to the unique sensory experiences of these infants in the NICUs, where they are exposed to various tactile stimuli, including both beneficial and potentially harmful interactions.

In addition, longitudinal studies suggest that preterm infants in the NICU may develop altered sensory responses as they grow. Children born extremely preterm showed reduced sensitivity to thermal sensations (hot and cold) by age 11, especially if they had undergone surgery as infants [21]. These changes suggest that early medical interventions may have lasting effects on sensory processing.

However, despite these challenges, studies have shown that early tactile interventions can improve sensory and emotional outcomes in preterm infants. In particular, kangaroo care has been shown to normalize autonomic function, improve oxygen saturation levels, and reduce stress markers such as cortisol [15,22,23]. In addition, preterm infants who receive consistent tactile stimulation through these interventions exhibit improved heart rate variability, reduced cortisol levels, and weight gain, critical indicators of healthy development [24]. In addition to supporting physiological stability, these interventions also promote emotional bonding between caregiver and infant, helping to offset the challenges of preterm birth [23].

## 3. Affective Touch: Definition and Mechanisms

*Affective touch* is a specialized tactile interaction characterized by gentle, slow, and rhythmic stroking movements. Typically performed at a speed of approximately 3 cm/s and often at a temperature close to that of human skin (~32 °C), this type of touch plays a unique role in emotional communication and bonding [7]. Unlike brief or functional tactile interactions, such as simple or discriminative touch, affective touch involves continuous movements. It is closely associated with positive emotional experiences, particularly in early caregiving contexts such as maternal caressing [13]. Affective touch differs from other forms of social touch, categorized as *simple*, *prolonged*, and *dynamic or affective touch* [13]. In particular, *simple touch* refers to brief and intentional contact during social interactions, while *prolonged touch* involves prolonged and often reciprocal physical contact, such as holding hands or hugging. On the other hand, affective touch is dynamic and repetitive and frequently involves stroking movements, which are particularly common in early caregiving interactions between mothers and infants [25].

The neurophysiological basis of affective touch involves a specialized set of nerve fibers called *C-tactile* (CT) afferents. The skin neural system, which represents the outermost boundary of the nervous system, serves as the primary sensor of environmental stimuli. It contains various afferent nerve fibers that transmit different types of sensory information to the brain, including information related to emotional experiences such as affective touch, pleasure, pain, heat, and fear [26]. This information is detected by a specific class of receptors, called *tactile receptors*, spread throughout the skin and specialized not only in the perception of physical stimuli but also in converting these stimuli into electrical signals [27]. These signals travel through the afferent nerve fibers of sensory neurons to the central nervous system, where they are processed and interpreted through a transduction mechanism [27]. When sensory signals with affective properties stimulate tactile receptors, they activate specific brain regions involved in emotion processing, contributing to the perception of pleasantness associated with touch [28]. Therefore, stimulating tactile receptors through affective touch may significantly strengthen social bonds [29].

Skin afferent fibers are classified according to their conduction speed, which is determined by the fiber’s thickness and degree of myelination [30]. Three main types of fibers have been identified: *Aβ fibers*, typically associated with discriminative touch, possess thick myelin sheaths that result in high conduction velocity, allowing rapid signal transmission from the skin to the central nervous system [31]; *Aδ fibers*, with thinner myelin sheaths, transmit information related to the perception of pain and temperature (both cold and hot) [32]; and *C-fibers*, thin and unmyelinated, conduct signals more slowly and are more sensitive to slow, low-force stimuli at neutral temperatures, such as a gentle caress [29].

CT afferents, a specialized subset of C-fibers, were first discovered in the hairy skin of mammals [33]. Since then, studies using microneurography have identified the presence of CT fibers in human skin, including in areas such as the face [34] and limbs [35]. CT fibers are predominantly found in hairy skin, while very few are found in glabrous skin (such as the palms). Due to the lack of myelination, CT afferents have slow conduction velocities, ranging from 0.6 to 1.3 m/s, which creates a delay between the initial perception of the stimulus and its arrival to the brain [36]. Their response is selectively activated by dynamic tactile stimuli applied with low force at a temperature close to the human skin, approximately 32 °C, and a low speed of approximately 3 cm/s, which is typical of a human caress [36,37]. Therefore, CT afferents act as “speed selectors,” responding only in a specific speed range for optimal activation [38].

A unique feature of CT afferents is their high fatigability, as they show a reduced response when identical stimuli are presented repeatedly [39], indicating that the CT fibers are finely tuned to respond to novel or emotionally significant tactile experiences rather than to repetitive and non-significant stimuli. Activation of CT afferents plays a crucial role in social touch, helping mediate affiliative behaviors and strengthen emotional bonds between individuals [29]. The “skin as a social organ” hypothesis further postulates that the emotional value of physical touch in social interactions reflects a mechanism explicitly mediated by the CT system, promoting social bonds [13].

The activation of CT afferents plays a critical role in the brain’s processing of tactile stimuli, particularly in the insular cortex, which is involved in assigning emotional value to sensory experiences [25]. While discriminative touch is processed in the somatosensory cortex, which is responsible for analyzing texture, position, and pressure, CT fibers project to the posterior insula, where the emotional meaning of touch is processed, allowing individuals to perceive it as pleasant or comforting [40,41]. The insula also plays a prominent role in broader social bonding mechanisms, as demonstrated by the activation of this region when mothers observe or interact with their infants, further highlighting its role in mediating emotional and affiliative responses to touch [42].

Importantly, research has shown that activation of CT afferents by affective touch also has far-reaching implications in neurohormonal processes that facilitate social bonding and emotional regulation. In particular, one of the critical mechanisms by which CT afferents exert their effects is the release of oxytocin, a neuropeptide widely recognized for its role in promoting social bonding, trust, and stress reduction [43]. When CT fibers are stimulated by gentle and slow touch, such as the caresses typical of nurturing interactions, oxytocin is released both peripherally, in response to skin stimulation, and centrally, in the brain [44]. This dual release contributes to the calming and bonding effects often associated with physical contact, particularly in emotionally significant contexts, such as mother–infant interactions. Oxytocin release in response to affective touch enhances emotional regulation by engaging brain regions involved in reward and social emotion processing. As seen above, these regions include the orbitofrontal cortex and insula, which are activated during CT fibers stimulation [45]. This neural circuitry suggests that oxytocin may act to enhance the emotional significance of social touch, making it perceived as more pleasurable and emotionally rewarding [41].

In addition, the effects of oxytocin also influence how individuals perceive and respond to touch depending on the social context. Studies have shown that oxytocin modulates responses to touch based on factors such as the familiarity or emotional closeness of the person providing the touch [46,47]. For example, the gentle touch of a loved one or caregiver is more likely to elicit positive emotional responses and higher levels of oxytocin release than the touch of a stranger. This contextual sensitivity is essential in early development, as it helps children distinguish between familiar and unfamiliar social partners, facilitating the formation of secure attachment and social learning [48]. The release of oxytocin during skin-to-skin contact or other forms of affective contact is significant for infants because their emotional regulation and physiological stability are closely linked to interactions with caregivers. In the newborn period, oxytocin not only helps infants regulate stress responses but also promotes better physiological outcomes, such as improved heart rate variability and lower cortisol levels [49]. These benefits are amplified in preterm infants, who are often deprived of constant physical contact due to medical interventions [19]. In addition, the influence of oxytocin extends to long-term social and emotional development. In fact, children who consistently experience nurturing touch that stimulates oxytocin release are more likely to develop secure attachment patterns that serve as a foundation for healthy emotional and social functioning throughout life [50].

The discovery and study of CT afferents have undoubtedly contributed significantly to our understanding of the mechanisms underlying affective touch. However, while the “affective touch hypothesis” originally proposed that CT afferents were primarily responsible for transmitting the pleasurable aspects of touch, more recent research has shown that not all pleasurable touch experiences are mediated by CT fibers. In fact, some experiences of affective touch may also involve Aβ fibers or other sensory pathways, suggesting that the relationship between CT fibers and affective touch is more complex than previously thought [25]. Furthermore, stimulation of CT fibers was found to be variable and influenced by several factors, including temperature, texture, and even emotional closeness to the person providing the touch, suggesting that affective touch is a complex and dynamic process [51].

Based on its neurobiological foundations, several studies have investigated the effects of affective touch on children’s emotional, social, and cognitive development using various physiological, behavioral, and neuroimaging methods (see Table 1).

Affective touch is essential not only for emotional regulation but also for fostering mother–infant bonding. Studies show that it plays a central role in developing secure attachment relationships. A caregiver’s gentle and nurturing touch can modulate an infant’s physiological stress response, including heart rate and cortisol levels [56]. In addition, affective touch helps regulate the autonomic nervous system, promoting parasympathetic regulation and improving infants’ resilience to stress [12]. 

In addition to its more immediate emotional effects, affective touch is thought to enhance social learning and attention in children. Carnevali et al. [57] showed that children who experienced affective touch could recognize and attend to complex visual stimuli, such as faces, suggesting that touch may facilitate cognitive processes related to social interaction. Furthermore, touch afferents have been shown to play a key role in reinforcing social behaviors and promoting affiliative bonding, highlighting their developmental importance in developing interpersonal connections [58].

Finally, recent research has expanded our understanding of the profound influence of affective touch on neurodevelopment through epigenetic mechanisms. In particular, studies have shown that early tactile interactions, such as maternal touch, play a critical role in DNA methylation, a process that regulates gene expression and influences long-term behavioral outcomes [55]. Maternal caregiving behaviors, including affectionate touch, have been associated with epigenetic changes in genes involved in stress regulation, such as the glucocorticoid receptor gene (NR3C1) and the oxytocin receptor gene (OXTR) [59,60]. These changes can protect infants from the adverse effects of stress and improve emotional regulation, suggesting that touch acts as a biological protective mechanism in early life [61]. Furthermore, touch-based interventions, such as skin-to-skin contact, have been shown to promote the restoration of DNA methylation patterns disrupted by the stress of preterm infants in NICUs, further supporting the role of tactile stimulation in epigenetic protection [62].

## 4. Preterm Birth and Affective Touch

As discussed in previous sections, affective touch is critical in promoting infants’ emotional regulation, bonding, and cognitive development. However, preterm infants, defined as those born before 37 weeks of gestation, face significant developmental challenges that affect their ability to benefit from such tactile stimulations fully. The rate of preterm birth remains high worldwide, with approximately 15 million infants born prematurely each year [52]. For these infants, the last weeks of gestation, which are critical for sensory and neurological development, are interrupted, resulting in incomplete development of sensory systems. Therefore, premature birth deprives infants of multisensory inputs in utero, including the important maternal touch that usually helps their nervous systems mature [7].

Premature infants often experience hypersensitivity to touch as a direct result of the immaturity of their sensory pathways [20]. This heightened sensitivity can make even the most delicate stimuli barely tolerable, contributing to increased stress responses. The environment of NICU, where many premature infants spend long periods, exacerbates these sensory challenges. Indeed, the NICU is often characterized by bright lights, loud noises, and frequent medical interventions, such as intubation or blood draws, which can be distressing for the infant [17,52]. These stimuli greatly contrast with the gentle and nurturing touch typically experienced by term infants in natural care environments. In addition, the NICU environment often results in reduced contact with primary caregivers because the infant is physically separated from caregivers for medical reasons, limiting opportunities for skin-to-skin contact [63].

This reduction in affective and nurturing touch in the NICU has profound implications for the emotional and cognitive development of preterm infants. As previously discussed, research has shown that affective touch, characterized by slow and gentle stroking that activates CT afferents, is essential for emotional regulation and bonding [7]. When preterm infants are deprived of this type of contact, they may experience delays in developing secure bonds with their caregivers, with negative consequences, as secure attachment is critical for a child’s emotional health and promotes resilience and social engagement as they grow [11,19]. Thus, without sufficient affective contact, preterm infants are at increased risk for developing emotional regulation difficulties, such as increased irritability and anxiety, as well as social withdrawal [1].

Moreover, preterm infants who lack affective touch often experience delays in cognitive development. Tactile stimulation is essential in brain development by promoting neuroplasticity and supporting the maturation of sensory processing brain areas, including the insula and somatosensory cortex [45]. As we have seen, these areas are critical in regulating not only sensory input but also emotional and cognitive functioning [64]. Therefore, the absence of consistent affective contact can lead to impaired neurodevelopment, resulting in long-term cognitive deficits, such as poor attention regulation, memory problems, and difficulties in social interactions [65,66]. Research on heart rate variability (HRV) in preterm infants also highlights the importance of touch for the autonomic nervous system in regulating stress responses. Studies show that dynamic and affective touch significantly improves HRV by increasing parasympathetic nervous system activity, promoting a calming effect, and supporting physiological regulation [19,52]. In contrast, lack of affective contact or exposure to non-caring contact can lead to increased stress responses and long-term dysregulation of physiological states. As a result, preterm infants who do not receive adequate tactile stimulation may be at greater risk for stress-related health problems and developmental delays later in life [19,67].

Premature infants face unique developmental challenges due to their early exposure to external stimuli and the limited availability of nurturing contact in the NICU. These infants require targeted interventions prioritizing affective contact to support their emotional, cognitive, and physiological development. Indeed, without sufficient opportunities for affective contact, the long-term developmental trajectories of preterm infants are at risk of significant impairment. In the following section, we will examine specific interventions, such as kangaroo care, aiming to reintroduce skin-to-skin contact as a key tool for improving developmental outcomes in preterm infants.

## 5. Skin-to-Skin Contact and Kangaroo Care for Preterm Infants

A significant practical application of the knowledge and understanding of the importance of tactile stimulation for infants, both term and especially preterm, is the use of *Skin-to-Skin Contact* (SSC) and its structured application in *Kangaroo Care* (KC). SSC is a method that uses the bare skin of the caregivers (usually the mother) and their presence to stabilize the infant. The contact must be skin-to-skin; therefore, the infant’s skin must be in direct contact with the caregiver’s skin. This intervention creates a sensory environment rich in positive stimulation, in which infants can experience the caregiver’s warmth, smell, and heartbeat, contributing to their physiological and emotional regulation [68]. SSC is crucial for preterm infants who are deprived of primary tactile, thermal, and auditory stimuli due to early separation from the womb. In particular, SSC has been identified as a critical factor in promoting healthy neuropsychological development in the preterm infant, as it provides external sensory stimulation that mimics the intrauterine environment, which helps to promote emotional bonding and regulate the infant’s physiological functions [63]. The principle behind SSC is that direct contact between the caregiver’s skin and the infant promotes activation of the CT afferents mentioned above, which are responsible for processing affective touch and activating emotion and stress regulatory pathways [7].

KC is a more structured and practical application of the principles of SSC, designed primarily for preterm or low-birth-weight infants. First developed in Colombia in the 1970s as a solution to the lack of incubators, KC involves a combination of continuous SSC, breastfeeding on demand, and early discharge from the hospital when appropriate [69]. While SSC can be practiced in any setting, KC is more formally integrated into neonatal care, where preterm infants are held upright against the caregiver’s chest for extended periods [69]. KC consists of three basic elements: early, continuous, and prolonged SSC between mother and infant; exclusive breastfeeding or formula feeding; and early discharge from the hospital once KC is established, with continued practice at home [70]. These components are supported by appropriate follow-up care at home, ensuring KC’s continuity and effectiveness. The World Health Organization (WHO) has recognized KC as an effective intervention for reducing neonatal mortality and improving long-term outcomes in preterm infants, particularly in low-resource settings [68]. Although both SSC and KC share the basic principle of promoting close and continuous contact, KC focuses explicitly on the therapeutic role of SSC in neonatal care, making it a formalized medical practice to promote the health and development of vulnerable infants [68].

KC is usually initiated when the clinical conditions of the preterm infant have stabilized. Nurses need to identify when the mother feels safe and gradually guide and support her through the process, which also involves the partner or other person chosen by the mother [68]. The introduction of KC begins with familiarizing the mother with the correct infant positioning. Over time, this process expands to include activities such as bathing, cord management, and safe handling of the newborn [68].

In the traditional KC position, the naked infant is placed between the mother’s breasts in an upright position, covered by a blanket or the mother’s clothing, as if wrapped in a baby carrier. This position allows the newborn to benefit from direct contact with familiar sounds, such as the mother’s voice and heartbeat, and scents reminiscent of the intrauterine environment, promoting an intimate reconnection with these sensory experiences of pregnancy. Several key steps must be followed to ensure proper implementation of KC [68]:

1. The caregiver, usually the mother, wears a loose-fitting gown tied in the front after she undresses to remain shirtless.
2. The caregiver sits in a comfortable chair and prepares to relax and receive the infant.
3. The infant, wearing only a diaper, hat, and socks to keep the extremities warm, is placed upright between the mother’s breasts.
4. The infant’s head is turned to the side so that one ear can rest on the mother’s chest, where the heartbeat can be heard.
5. The infant’s legs and arms are frog-folded, mimicking the fetal position.
6. The infant’s abdomen is directly against the mother’s chest.
7. The mother supports the baby’s bottom with one hand and covers it with her gown or a blanket to provide warmth and protection.

SSC and KC have been shown to have significant physiological benefits for both term and preterm infants. One of the most important effects of SSC is its ability to promote thermoregulation [71]. Preterm infants, due to the immaturity of their skin and metabolic systems, are highly susceptible to hypothermia, which can lead to severe complications. During SSC, the caregiver’s body acts as a natural heater, adjusting its temperature to keep the infant’s temperature within an optimal range [72]. Specifically, studies show that SSC is more effective at maintaining stable body temperature than traditional incubators [18]. In addition, SSC and KC have been shown to stabilize heart rate and improve respiratory function, particularly in preterm infants [73]. Research suggests that the close contact that characterizes SSC helps synchronize the infant’s physiological rhythms with the caregiver, reducing bradycardia, apnea, and respiratory irregularities [74].

In addition to these immediate benefits, SSC has been shown to increase oxygen saturation, reduce the need for mechanical ventilation, and promote more significant weight gain in preterm infants. These improvements are partly due to the infant’s reduced stress response during SSC, which promotes better nutrient absorption and energy conservation [69,75]. Furthermore, it has been demonstrated that preterm infants who received regular SSC and KC had significantly fewer nosocomial infections, shorter hospital stays, and better overall outcomes than those who did not receive these interventions [76]. In addition, breastfeeding rates were significantly higher among infants who received KC, as SSC promotes early breastfeeding behavior and stimulates lactation in mothers [77,78].

The emotional and neurophysiological benefits of SSC and KC are equally significant. For preterm infants, who are often exposed to sensory deprivation and stress in the NICUs, the SSC provides a welcoming environment where the infant can experience rhythmic and soothing stimuli that mimic those in the womb. Research shows that the SSC reduces the infant’s cortisol levels, which promotes emotional regulation and reduces signs of distress [79]. In addition, the caregiver’s warmth, voice, and heartbeat provide comforting stimuli by activating the infant’s parasympathetic nervous system and releasing oxytocin. As discussed above, oxytocin is essential because it is a hormone associated with social bonding and stress reduction [44] and it plays a key role in establishing secure attachment, which is critical for a child’s long-term emotional and social development [48].

SSC and KC also promote appropriate neurodevelopment. Early tactile stimulation through affective touch activates brain plasticity and promotes the maturation of neural circuits involved in sensory processing and emotional regulation [65]. Studies using neuroimaging techniques show that children who regularly receive SSC have increased neural activity in regions such as the insula, orbitofrontal cortex, and prefrontal cortex, which are essential for integrating sensory input and regulating emotions [80]. These regions are responsible for affective touch processing and are integral to developing social cognition and emotional resilience. In particular, preterm infants who receive consistent tactile stimulation according to the parameters of affective touch are more likely to achieve developmental milestones related to attention, memory, and social interaction than those who do not [10,57].

In addition to these cognitive benefits, the stress-reducing effects of SSC and KC are significant in preterm infants, who are vulnerable to increased stress responses caused by medical interventions and the NICU environment [12]. Reducing stress responses through SSC contributes to long-term improvements in self-regulation and emotional control, laying the foundation for better mental health and social functioning later in life [68]. In addition, premature infants regularly exposed to SSC and KC show improved academic performance and social adaptability in childhood and adolescence, reflecting the lasting impact of early tactile interventions on brain development [81].

## 6. Practical Implications and Future Research Directions

Research on affective touch and its role in promoting neurodevelopment and emotional regulation in infants has important practical implications for neonatal care, particularly for preterm infants. One of the most important implications is prioritizing tactile stimulation as a critical component of caregiver–newborn interactions. Encouraging caregivers to engage in gentle, reassuring touch activities can promote and enhance attachment bonding, reduce infant stress responses, and support infant cognitive and emotional development [82]. This practical implication is significant for preterm infants, as the lack of natural tactile stimulation in the NICU environment can impair neurodevelopment. Therefore, neonatal care protocols should emphasize the importance of integrating affective touch into daily care routines, either through direct contact with caregivers or therapeutic interventions designed to replicate this sensory experience [83].

Interventions that are most effective in promoting affective contact include SSC and massage therapy. SSC, as previously discussed, provides infants with continuous contact that stabilizes physiological functions such as heart rate and breathing while promoting emotional attachment [79]. Massage therapy involves stroking the infant’s body and applying gentle pressure, activating CT fibers that contribute to emotional regulation and reducing stress. Research has shown that preterm infants who receive regular massage therapy have better weight gain, lower cortisol levels, and improved sleep patterns than those who do not [16,84].

In addition to the interventions described, recent research highlights the critical role of sensorimotor and physical interventions in improving both developmental outcomes and parent–child relationships in preterm infants. Girolami and colleagues have shown that structured early intervention programs that focus on tactile and kinesthetic stimulation can significantly improve the motor skills of preterm infants, who are often at risk for motor delays [85]. By involving parents in the interventions, these physiotherapy programs not only promote motor development, such as postural control and selective movement but also strengthen the parent–child bond, which is essential for reducing parental stress and promoting healthy early interactions. Through hands-on activities, parents are taught to be active participants in their child’s development, promoting a nurturing environment that can mitigate some of the challenges associated with preterm birth [86,87]. Complementing this perspective, Fucile and colleagues demonstrated that combined oral and non-oral sensorimotor interventions improve not only growth and motor function [88] but also oral feeding skills in preterm infants by facilitating the complex coordination required for efficient feeding [89]. This multisensory approach significantly reduced the time required to achieve independent oral feeding compared to single-modality interventions, suggesting a synergistic effect of multisensory inputs [90,91].

These findings highlight the potential benefits of a comprehensive tactile approach to support preterm infants in the development of essential motor skills, thereby improving short- and long-term developmental outcomes. These interventions, particularly in the NICU, can provide the necessary sensory input that preterm infants are deprived of due to medical interventions and the clinical environment. Furthermore, they are cost-effective and can be easily incorporated into neonatal care protocols [92].

Future clinical directions should focus on developing standardized protocols promoting affective touch in neonatal care. Although SSC and massage therapy are well-established interventions, there remains a need to explore additional methods of incorporating tactile stimulation into routine care, particularly for infants who cannot experience direct SSC due to their medical conditions. Training healthcare personnel to recognize the importance of touch and equipping them with strategies to incorporate affective touch into their care routines can significantly impact the developmental outcomes of preterm infants [83,93,94]. In addition, empowering caregivers to engage in affective touch practices, both in the NICU and at home, will be critical to the long-term developmental success of infants [95].

Despite the growing body of research on these topics, there are still gaps in the literature on affective touch and preterm infant development. While much is known about the immediate benefits of SSC and massage therapy, the long-term effects of affective touch on the neurodevelopmental trajectories of preterm infants remain poorly understood. Thus, further research is needed to examine how early tactile experiences influence preterm infants’ cognitive, emotional, and social development during childhood and adolescence. In addition, there is a limited understanding of how variations in tactile stimulation, such as frequency and duration, influence outcomes, highlighting the need for studies that examine these variables in clinical settings [19,52].

Future research should also explore new methods to study affective touch and C-fiber function in developmental clinical settings. Advanced imaging techniques, such as functional magnetic resonance imaging (fMRI) and diffusion tensor imaging (DTI), could study how touch-related neural pathways develop in preterm infants and relate to emotional regulation and cognitive outcomes. In addition, wearable technologies could be used to monitor infants’ physiological responses to affective touch, providing real-time data that could help tailor interventions to the needs of individual infants. These innovations would allow for more precise measurement of the effects of tactile stimulation on infant development, bridging the gap between theory and practice [7].

In light of research on early tactile experiences and epigenetics, an area that certainly needs to be explored is the long-term effects of affective touch on the epigenome and how these early tactile experiences may influence neurodevelopmental trajectories across the lifespan. Indeed, exploring the links between neuroscience, behavioral genetics, and developmental psychology may shed light on how to optimize haptic interventions to support vulnerable populations, particularly preterm infants, in both clinical and home care settings [96]. Understanding the epigenetic effects of caregiver touch will be key to developing personalized interventions that harness the power of affective touch to improve developmental outcomes in vulnerable infants.

Finally, there is a strong need for interdisciplinary research that integrates neuroscience, developmental psychology, and pediatrics. The complex relationship between sensory input, brain development, and emotional regulation in preterm infants cannot be fully understood by one discipline alone. Collaboration among researchers, clinicians, and psychologists will provide a more comprehensive understanding of how affective touch contributes to the long-term developmental outcomes of preterm infants. Integrating neuroscience and developmental psychology findings into pediatric care protocols will help ensure that neonatal care practices are based on the latest scientific evidence, ultimately improving the well-being of preterm infants.

## 7. Conclusions

In conclusion, this review has highlighted and confirmed the fundamental role of affective touch in early development, especially for preterm infants. From the neurobiological mechanisms underlying affective touch to its emotional and cognitive benefits, it is clear that tactile interactions between caregivers and infants are essential for regulating physiological states, promoting attachment, and supporting long-term developmental outcomes. Affective touch not only facilitates the formation of secure attachment but also plays a crucial role in promoting self-regulation and resilience to stress, which are critical for both term and preterm infants.

Interventions such as SSC and KC are effective in bridging the gap between preterm babies and the nurturing environment they are deprived of in NICUs. As detailed in the review, these interventions promote significant physiological benefits (improvements in heart rate, breathing, and temperature regulation) while promoting the development of a positive attachment bond between caregiver and infant. The evidence reviewed emphasizes that constant tactile stimulation in the form of affective touch, particularly in the NICU, is crucial for promoting vulnerable infants’ cognitive, emotional, and social development.

In the future, neonatal care practices will increasingly need to incorporate these findings to ensure that interventions based on early tactile stimulation are part of standard care for preterm infants. Incorporating affective touch into clinical protocols, caregiver training, and education can significantly improve developmental outcomes in vulnerable populations. Future research should focus on understanding the long-term effects of affective touch, including its epigenetic influences, and exploring how innovative approaches can further optimize these tactile interventions. By integrating the interdisciplinary knowledge of neuroscience, developmental psychology, and pediatrics, it will be possible to ensure that neonatal care evolves to support the well-being of all infants, particularly those born prematurely, at increased risk of adverse developmental outcomes and difficulties in later life.

## Figures and Tables

**Table 1:** Overview of methodologies used to study the role of affective touch in infant development.

Methodology	Examples of Measurements	Purpose	Applications
Physiological Measures [12,19,52]	Heart rate variability (HRV) Oxygen saturation	Assess autonomic response to affective touch interventions	Increased HRV and oxygen saturation indicate stress reduction and improved autonomic regulation
Neuroimaging Techniques [53,54]	High-density diffuse optical tomography (HD-DOT) Functional near-infrared spectroscopy (fNIRS) Electroencephalogram (EEG)	Observe cortical activation in areas associated with sensory and affective processing	Activation in the insula in response to CT-afferent stimulating touch (e.g., slow, gentle strokes)
Behavioral Observation Paradigms [12,55]	Still face paradigm	Assess social engagement, emotional regulation, stress resilience	Infant visual and physical responses to maternal touch
Questionnaire-Based Assessments [54]	Maternal mental health questionnaires (e.g., Edinburgh Postnatal Depression Scale)	Account for maternal factors influencing touch interactions	How maternal well-being affects the quality and frequency of touch

## Data Availability

No new data were created or analyzed in this study. Data sharing is not applicable to this article.
